# Interstitial Lung Disease as a Herald of P-ANCA Vasculitis: A Case of Evolving Multisystem Disease

**DOI:** 10.7759/cureus.87536

**Published:** 2025-07-08

**Authors:** Tamiru B Berake, Shray Ambe, Lense Negash, samuel Gretz, Babray Laek

**Affiliations:** 1 Internal Medicine, Tufts Medical Center, Boston, USA; 2 Internal Medicine, New York Medical College, Metropolitan Hospital Center, New York, USA; 3 Nephrology, Tufts Medical Center, Boston, USA; 4 Rheumatology, Tufts Medical Center, Boston, USA

**Keywords:** antineutrophil cytoplasmic antibody (anca) associated vasculitis (aav), diffuse alveolar hemorrhage, glumerulonephritis, inflammatory myositis, intrestitial lung disease

## Abstract

We present the case of a 72-year-old man with idiopathic pulmonary fibrosis (IPF) who presented with stiffness and pelvic girdle pain and was initially treated for polymyalgia rheumatica (PMR). Despite transient improvement on steroids, persistent symptoms and atypical MRI findings led to muscle and renal biopsies, revealing necrotizing vasculitis and pauci-immune glomerulonephritis. Positive myeloperoxidase and perinuclear antineutrophil cytoplasmic antibody titers confirmed the diagnosis of antineutrophil cytoplasmic antibody-associated vasculitis (AAV). The patient's course was complicated by diffuse alveolar hemorrhage requiring intubation, which improved with pulse steroids and cyclophosphamide. The patient stabilized on methotrexate and methylprednisolone. This case highlights the complexity of diagnosing AAV, especially with overlapping PMR-like symptoms and underlying interstitial lung disease.

## Introduction

Antineutrophil cytoplasmic antibody (ANCA)-associated vasculitides (AAV) are systemic diseases affecting small- to medium-sized vessels, often involving the kidneys, lungs, skin, and musculoskeletal system [1]. Myeloperoxidase (MPO)-positive perinuclear antineutrophil cytoplasmic antibodies (P-ANCA) are most commonly associated with microscopic polyangiitis, but they can also be found in granulomatosis with polyangiitis and eosinophilic granulomatosis with polyangiitis [2]. We report a diagnostically challenging case of P-ANCA vasculitis initially treated as PMR with eventual multisystem involvement.

## Case presentation

A 72-year-old man with a history of hypertension and idiopathic pulmonary fibrosis (IPF) (diagnosed two years earlier and managed with pirfenidone) presented with progressive bilateral muscle pain, fatigue, and stiffness of four months’ duration. Laboratory evaluation (Table 1) revealed elevated inflammatory markers (erythrocyte sedimentation rate [ESR] and C-reactive protein [CRP]) and a mild elevation in creatine kinase. Based on age, pelvic girdle involvement, stiffness, and elevated inflammatory markers, a presumptive diagnosis of polymyalgia rheumatica (PMR) was made, and prednisone therapy was initiated.

**Table 1:** Laboratory summary C-ANCA, cytoplasmic antineutrophil cytoplasmic antibody; GFR, glomerular filtration rate; HMGCR, 3-hydroxy-3-methylglutaryl-CoA reductase; MPO, myeloperoxidase; P-ANCA, perinuclear antineutrophil cytoplasmic antibody

Test	Value	Reference Range	Units
Creatinine	1.63	0.5–1.2	mg/dL
Estimated GFR	45	>60	mL/min/1.73 m²
Creatine kinase	183	41–331	U/L
C-reactive protein	171	0–10	mg/L
Erythrocyte sedimentation rate	107	0–30	mm/hr
P-ANCA	1:80	<1:20	Titer
C-ANCA	1:20	<1:20	Titer
Anti-MPO antibody	>200	<20	U/mL
Anti-PR3 antibodies	<0.2	0–0.9	U/mL
Anti-HMGCR antibody	<20	<20	U/mL
White blood cell count	10.1	3.4–10.8	×10³/µL
Platelets	284	150–450	×10³/µL
Hemoglobin	10	13–17.5	g/dL
Calcium	9.4	8.5–10.5	mg/dL
Aspartate aminotransferase	12	6–42	U/L
Alanine aminotransferase	9	6–55	U/L

**Figure 1:**
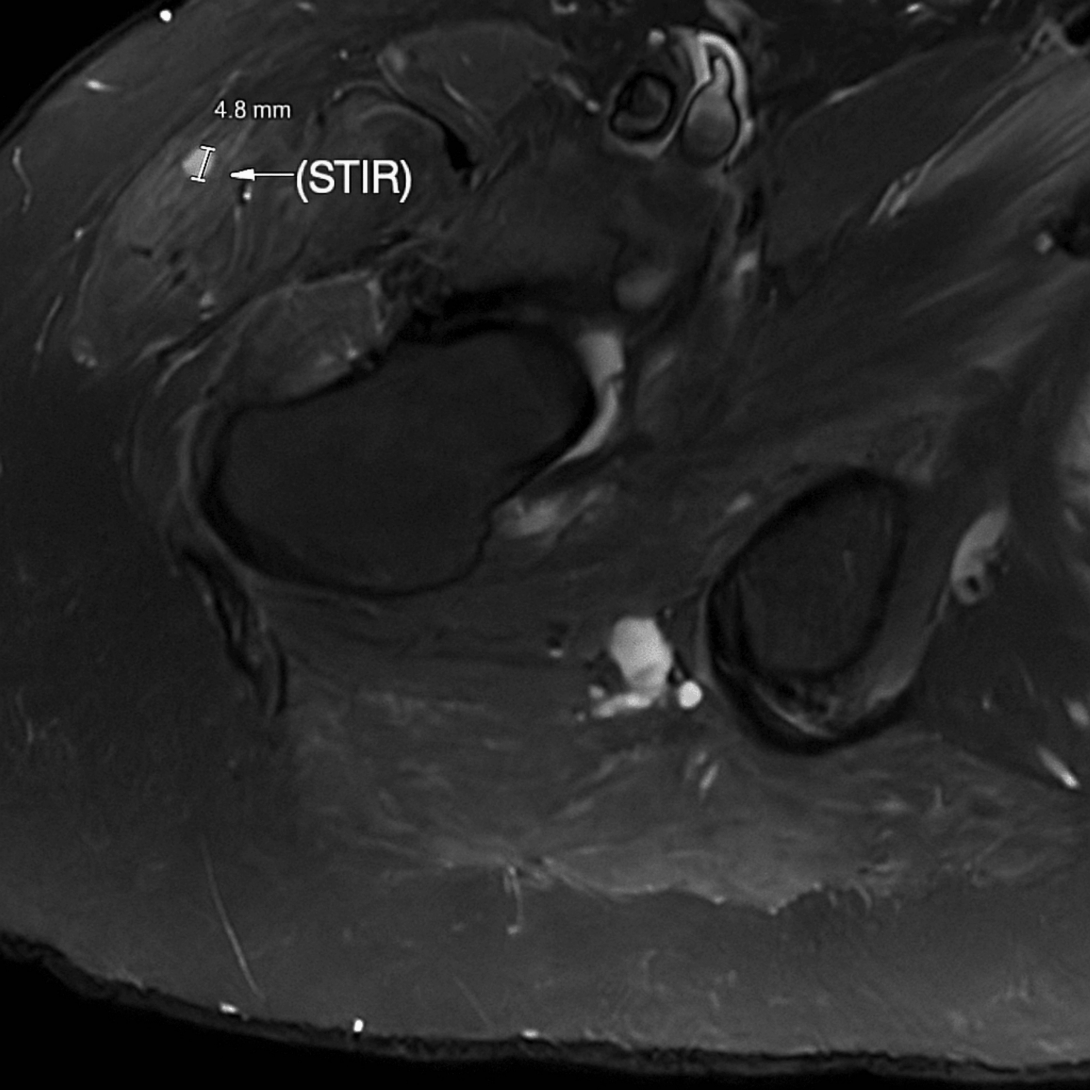
Axial STIR MRI of the right thigh Arrow showing diffuse hyperintensity and edema in the adductor muscles, consistent with inflammatory myopathy. STIR, short-tau inversion recovery

**Figure 2:**
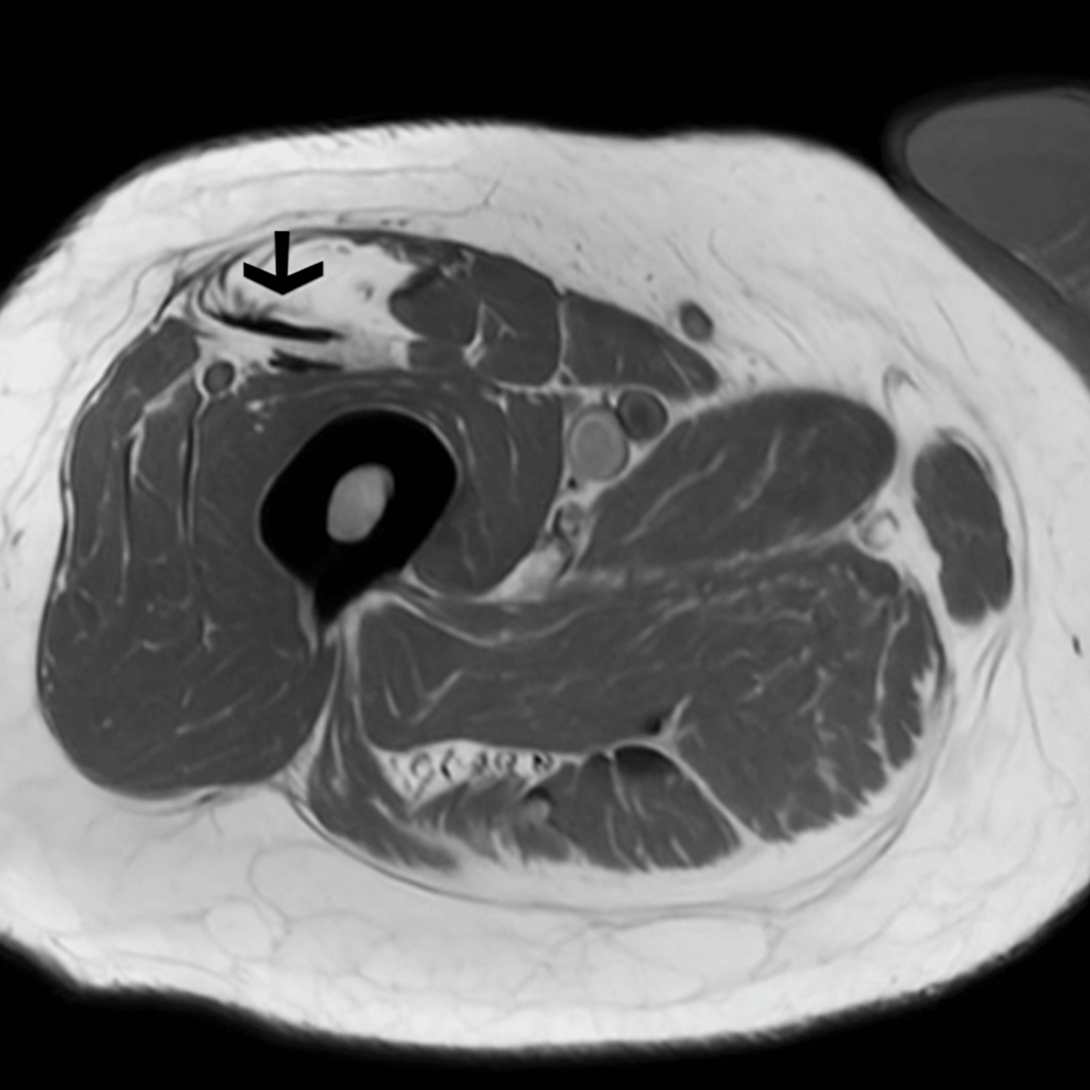
Axial T1-weighted MRI of the right thigh MRI of the right thigh showing fatty infiltration(arrow) of the rectus femoris and muscle atrophy

However, due to persistent thigh soreness and recurrence of symptoms despite steroid therapy, an MRI of the right femur was obtained. Imaging (Figures 1, 2) revealed moderate edema in the medial adductor musculature, fatty infiltration of the rectus femoris, and diffuse multifocal 3- to 7-mm foci of short-tau inversion recovery (STIR) hyperintensity and enhancement throughout the proximal thigh. These findings were atypical for PMR and raised concern for other autoimmune or infectious myositis rather than PMR. Two years prior, the patient was evaluated for shortness of breath. Pulmonary function tests showed preserved spirometry with reduced diffusion capacity for carbon monoxide (DLCO), and chest CT revealed reticular opacities, traction bronchiectasis, and focal honeycombing, findings consistent with chronic interstitial lung disease with a usual interstitial pneumonia pattern. A diagnosis of IPF was made, and the patient was started on pirfenidone.

Investigations

**Figure 3:**
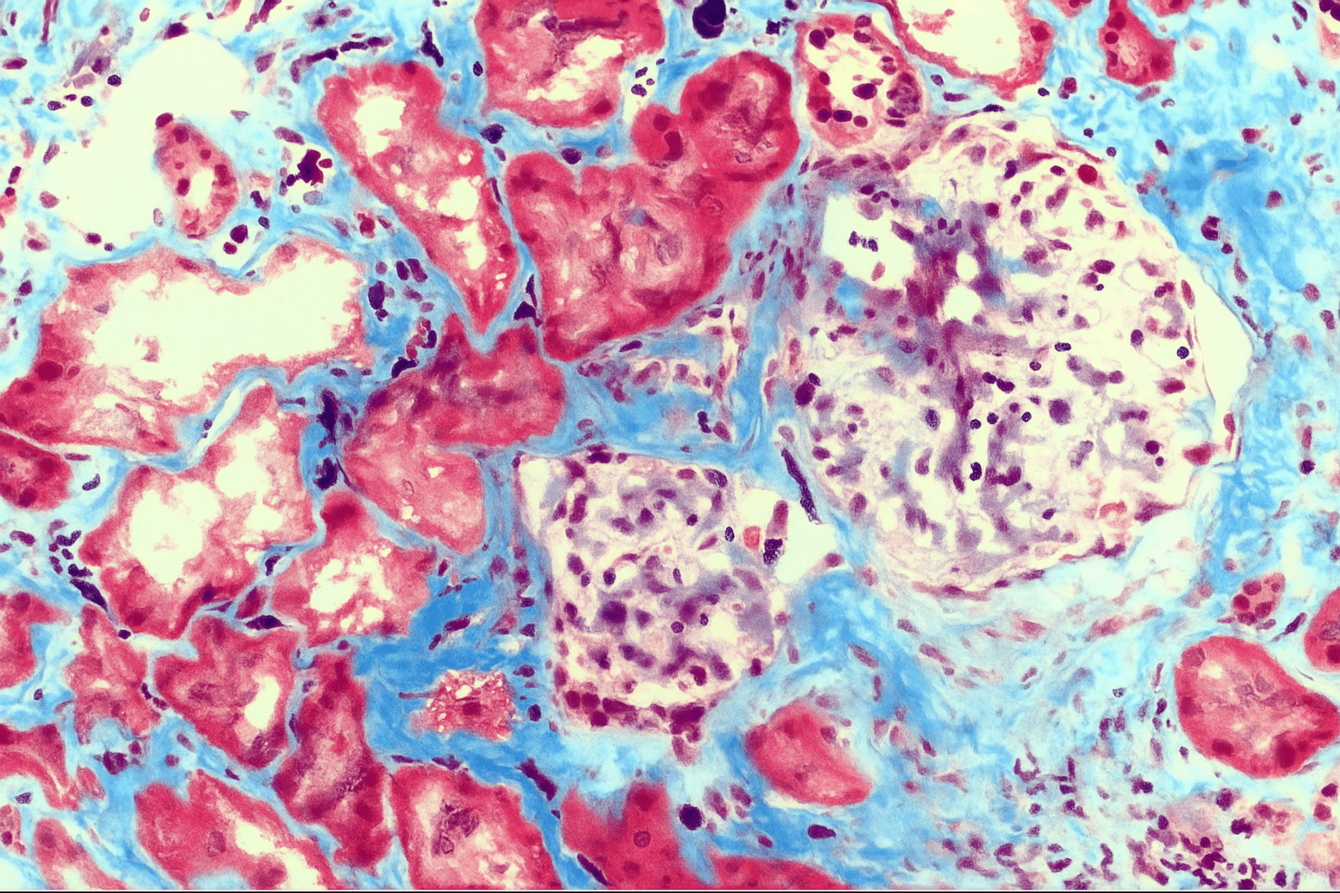
Renal biopsy Masson’s trichrome stain of kidney biopsy showing glomeruli with ischemic-type involution, interstitial fibrosis, and tubular atrophy without crescent formation.

**Figure 4:**
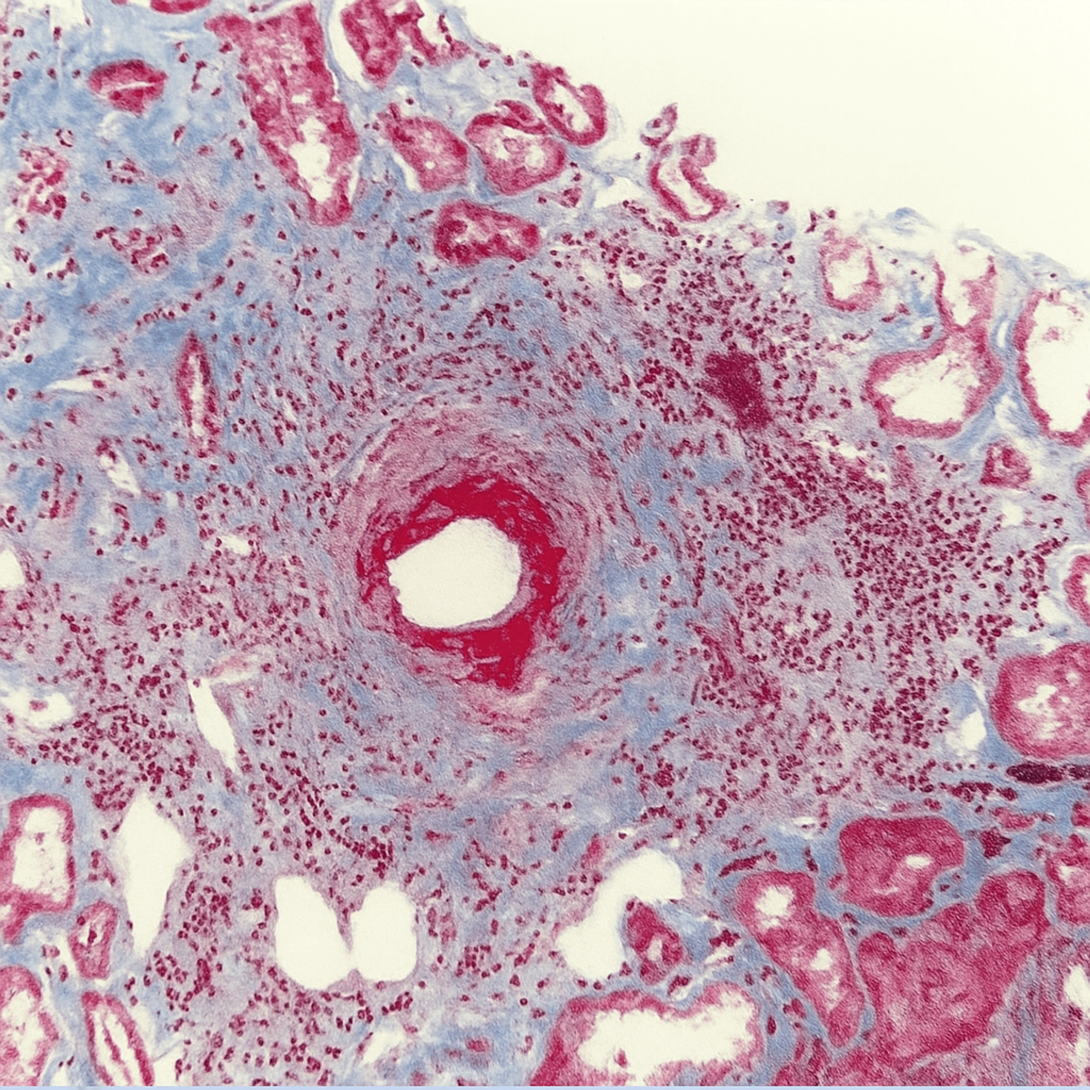
Renal biopsy Small artery with vasculopathic changes and perivascular inflammation on Masson's trichrome stain

Given the atypical imaging findings and incomplete clinical resolution, a biopsy of the right thigh muscle was performed. This revealed necrotizing granulomatous vasculitis involving small- to medium-sized vessels, shifting the working diagnosis toward systemic vasculitis (Figures 3, 4).

Serum testing demonstrated MPO positivity (greater than 200), p-ANCA at a titer of 1:80, and negative PR3. The patient’s full laboratory workup is summarized in Table 1, including serologies, inflammatory markers, and renal indices.

The patient was referred to nephrology after lab studies indicated worsening renal function, and urinalysis showed proteinuria and hematuria. A renal biopsy (Figures 3, 4) revealed pauci-immune glomerulonephritis with focal and global glomerulosclerosis, fibrinoid necrosis in some glomeruli, cellular and with no crescent formation, moderate interstitial fibrosis, and tubular atrophy, consistent with histologic findings of active P-ANCA-associated vasculitis.

In light of new-onset visual changes, a temporal artery biopsy was performed to evaluate for giant cell arteritis. The specimen demonstrated no evidence of active arteritis, with no inflammatory infiltrates.

Laboratory evaluation over time revealed progressive worsening of renal function (creatinine from 1.3 to 1.6 mg/dL), leukocytosis (WBC ~14 × 10⁹/L), anemia (hemoglobin 9.8-11.7 g/dL), and persistently elevated ESR and CRP. Urinalysis demonstrated proteinuria with urine protein-to-urine creatinine ratio (UPCR) peaking at 1,100 mg/g and red blood cell casts. Autoimmune and infectious workup was otherwise negative. Antinuclear antibody (ANA) test, hepatitis panel, 3-hydroxy-3-methylglutaryl-CoA reductase (HMGCR) antibody, and tuberculosis screening returned negative.

The patient was started on rituximab infusions and avacopan for induction therapy. In light of data supporting avacopan as a steroid-sparing agent in ANCA-associated vasculitis, corticosteroids were tapered. The patient was also started on prophylactic trimethoprim/sulfamethoxazole for *Pneumocystis jirovecii* pneumonia. His rheumatologist planned to continue rituximab maintenance for one to two years, with consideration of methotrexate for long-term disease-modifying therapy after remission. Six weeks in his treatment unfortunately, his course turned to the worst and he developed acute hypoxic respiratory failure. Bronchoscopy with lavage confirmed diffuse alveolar hemorrhage. He was intubated and initiated on pulse dose steroid with methylprednisolone 1 g and cyclophosphamide. His symptoms improved after receiving a pulse dose of methylprednisolone (1 g) and cyclophosphamide. At the time of manuscript submission, he was extubated and maintained on high-flow oxygen with methotrexate and intravenous methylprednisolone 60 mg, showing good respiratory recovery.

## Discussion

This case underscores the clinical challenge of differentiating PMR from other inflammatory myopathies and systemic vasculitides. The patient’s initial presentation was suggestive of PMR, but muscle MRI findings and histopathology ultimately pointed to P-ANCA vasculitis. Importantly, his interstitial lung disease, presumed idiopathic, may, in fact, represent pulmonary involvement from ANCA-associated vasculitis. The increasing recognition of interstitial lung disease (ILD) as a presenting or isolated manifestation of ANCA-associated vasculitis supports this interpretation [1].

The co-occurrence of PMR, ILD, and P-ANCA-associated vasculitis is rare but increasingly recognized in clinical practice. Several case reports and series have documented this triad, highlighting the diagnostic challenges and the importance of considering systemic vasculitis in patients with overlapping rheumatologic features [2,3]. The delay in diagnosis often stems from the elusiveness of early symptoms and the lack of specificity of initial laboratory findings, which can mimic more benign inflammatory conditions such as PMR.

This case highlights the evolving treatment landscape of ANCA-associated vasculitis. The incorporation of avacopan [4-7], a C5a receptor inhibitor, represents a significant advancement in reducing reliance on high-dose corticosteroids and mitigating their associated side effects. However, this case also illustrates that even with biologic intervention, patients may still develop life-threatening complications such as diffuse alveolar hemorrhage, underscoring the aggressive nature of the disease and the importance of close monitoring.

Our patient’s favorable response to cyclophosphamide and pulse-dose steroids after developing diffuse alveolar hemorrhage reaffirms the utility of traditional immunosuppressive regimens in managing severe disease flares. His recovery trajectory on maintenance therapy with methotrexate and moderate-dose steroids demonstrates the potential for disease control when treatment is individualized and escalated promptly.

Clinicians should maintain a high index of suspicion for systemic vasculitis in patients presenting with atypical or relapsing inflammatory symptoms or subtly multisystem involvement. Early biopsy and serologic evaluation are essential to avoid diagnostic delays and irreversible organ damage. Furthermore, interdisciplinary collaboration among rheumatology, nephrology, and pulmonology is crucial in managing such complex, multisystem diseases.

## Conclusions

This case illustrates the diagnostic complexity of ANCA-associated vasculitis, particularly when initial symptoms are subtle or involve isolated organ involvement. Early tissue diagnosis and comprehensive serologic workup are critical in identifying systemic vasculitis before irreversible organ damage occurs. While newer therapies such as avacopan offer steroid-sparing benefits, traditional immunosuppressive strategies remain essential in managing severe disease manifestations such as DAH. Interdisciplinary collaboration is crucial to enhancing outcomes in these complex cases.
